# Supplementary material for: Preclinical evaluation of tissue-selective gene therapies for congenital generalised lipodystrophy
Source: Gene Ther. 2024 Jul 28;31(9-10):445–54. doi: 10.1038/s41434-024-00471-z (PMC11399081; doi:10.1038/s41434-024-00471-z)
Supplement: Supplementary file 1 — Supplemental Figure 1 [file 41434_2024_471_MOESM1_ESM.pdf]

Preclinical evaluation of tissue-selective gene therapies for congenital generalised lipodystrophy

Mansi Tiwari[1,2], Ahlima Roumane[1,2], Nadine Sommer[1,2], Weiping Han[3,4,5], Mirela Delibegović[2,6], Justin J. Rochford[1,2] and George D. Mcilroy[1,2*]

Fig. S1 Tissue-selective gene therapy in lipodystrophy. A Graphical illustration of the physiological and metabolic measurements performed in 15- to 17-week-old male and female SKO mice. Relative gene expression levels of hepatic metabolic markers in the liver of AAV-aP2 (**B**) and AAV-TBG (**C**) injected mice eight weeks after I.P. injection of 1×10^{12} genome copies. **D** Tissue weights of retroperitoneal white adipose tissue (rWAT) from WT, AAV-CMV, AAV-aP2 and AAV-TBG injected mice. Relative gene expression levels of adipocyte markers in gWAT of AAV-aP2 (**E**) and AAV-TBG (**F**) injected mice eight weeks after I.P. injection of 1×10^{12} genome copies. **G** Western blot tissue panel analysis (excluding the liver) of human BSCL2 protein levels in AAV-TBG injected mice. **H** Western blot analysis of human BSCL2 protein levels in gWAT of AAV-CMV, AAV-aP2 and AAV-TBG injected mice. All data are biological replicates presented as the mean ± SD, n = 16 (WT), 5-6 (AAV-GFP), 5 (AAV-aP2) and 5 (AAV-TBG) mice per group for (**B**, **C**, **E, F)**, n = 44 (WT), 5 (AAV-CMV), 5 (AAV-aP2) and 5 (AAV-TBG) mice per group for (**D)**, *$p < 0.05$, **$p < 0.01$, ***$p < 0.001$ and ****$p < 0.0001$ vs. WT, $^{\#\#}p < 0.01$, $^{\#\#\#}p < 0.001$ and $^{\#\#\#\#}p < 0.0001$ vs. AAV-GFP, ntd no tissue dissected, $ not detected, L indicates the presence of a molecular ladder.

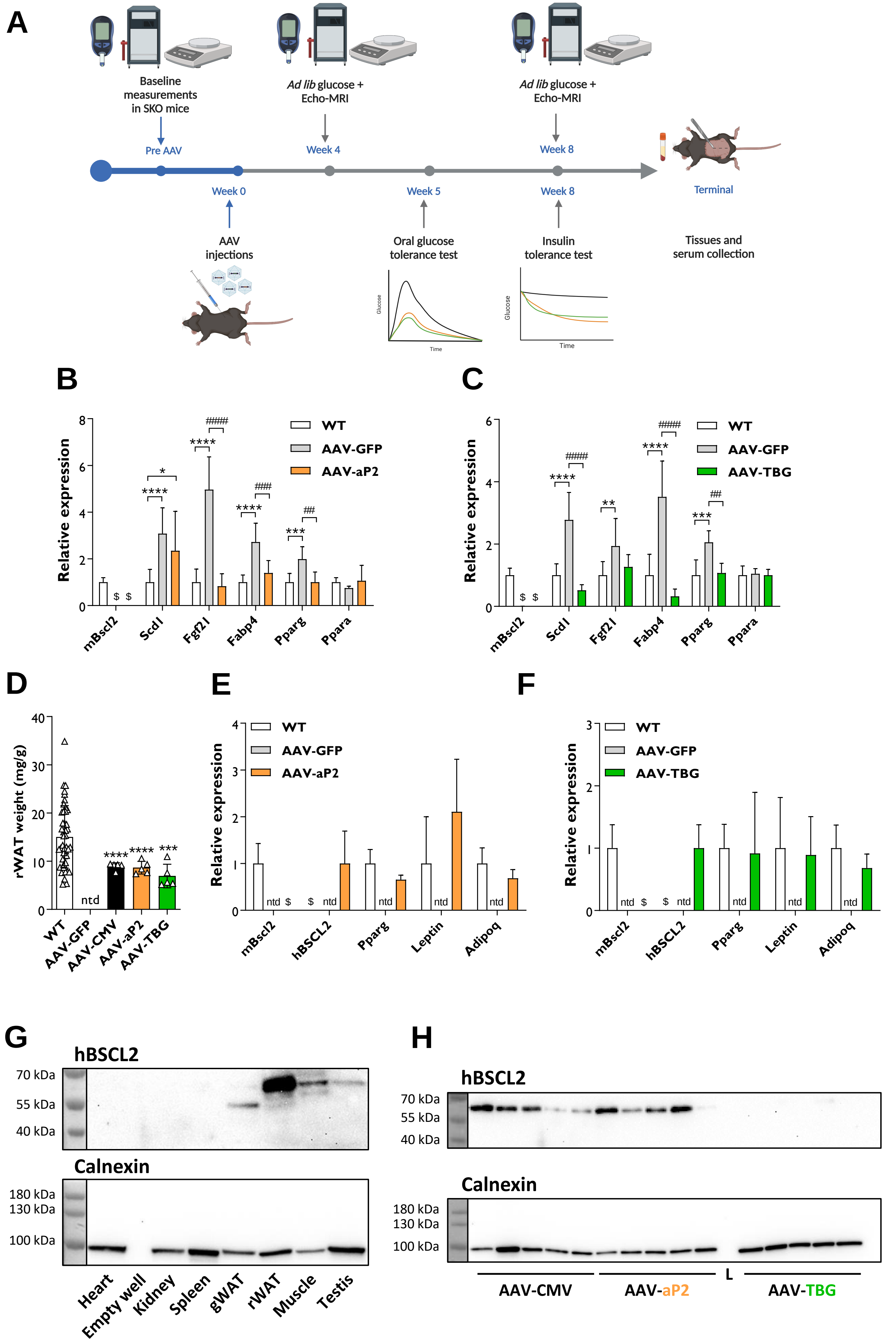

A
Baseline measurements in SKO mice
Pre AAV
Ad lib glucose + Echo-MRI
Week 4
Ad lib glucose + Echo-MRI
Week 8
Week 0
AAV injections
Week 5
Oral glucose tolerance test
Week 8
Insulin tolerance test
Terminal
Tissues and serum collection
Glucose
Time
B
Relative expression
WT
AAV-GFP
AAV-aP2
mBscl2
Scd1
Fgf21
Fabp4
Pparg
Ppara
C
WT
AAV-GFP
AAV-TBG
D
rWAT weight (mg/g)
ntd
WT
AAV-GFP
AAV-CMV
AAV-aP2
AAV-TBG
E
hBSCL2
Leptin
Adipoq
F
G
hBSCL2
70 kDa
55 kDa
40 kDa
Calnexin
180 kDa
130 kDa
100 kDa
Heart
Empty well
Kidney
Spleen
gWAT
rWAT
Muscle
Testis
H
L
AAV-CMV
AAV-aP2
AAV-TBG
